# Back to the bedside: the role of bedside teaching in the modern era

**DOI:** 10.1007/s40037-014-0111-6

**Published:** 2014-02-25

**Authors:** Zeshan Qureshi

**Affiliations:** UCL Institute of Child Health, 30 Guilford Street, London, WC1N 1EH UK

Fifty years ago, three-quarters of clinical teaching was at the bedside [1]. Today’s estimates are 8–19 % [2]. This reduced exposure in undergraduate years to a critical aspect of training may be responsible, in part, for the declining clinical skills of junior doctors [3]. Peters and ten Cate [4] have reviewed the literature on bedside teaching, assessing its strengths, the causes of its decline, and its potential role in medical education.

## Why has bedside teaching declined

Hospitals are now busier with significantly increased patient throughput. Teachers are required to see more patients, and fill in increasing amounts of paperwork with no additional time allocated for these tasks. Opportunity and time to teach at the bedside is more limited. Senior teachers may feel under-prepared for teaching students who have a radically different undergraduate course to the ones they experienced, whilst junior staff who may have a better grasp of the new-style curricula have limited teaching opportunities.

Bedside teaching is also in competition with other teaching modalities that are easier to plan, including rapidly developing clinical simulation techniques, often as part of clinical skills centres. Students feel more comfortable with the possibility of making mistakes around simulated patients. They also have the advantage of direct feedback from the actor, and the opportunity for repetition of difficult scenarios.

There has been a paradigm shift in clinical diagnosis. There is an increasing dependence on sophisticated technology and laboratory tests rather than clinical examination skills fostered at the bedside. This philosophy has resulted in a partial shift of ward rounds from the bedside to the conference room, where bedside interaction is sacrificed for better access to complex imaging and laboratory test results.

There may also be a perception that the bedside teaching is demeaning to patients, interestingly more commonly amongst junior staff. However, Peters and ten Cate report that 77–85 % of patients enjoy being part of bedside teaching [4–7]. Harm is avoided by ensuring adequate training of teachers, consenting patients properly, and respecting patient dignity.

Interestingly, Peters and ten Cate mention the poor clinical skills of medical students as a practical hindrance to bedside teaching. However, I would argue that this gives even greater justification for expanding bedside teaching programmes, where such skills were historically learned.

## Benefits of bedside teaching

Bedside teaching is strongly supported by both students and teachers. Students gain first-hand experience of the doctor–patient relationship. ‘Patient-centred’ care is directly observed and learned. The student experiences not just how to assess disease, but beyond this, how to personally and professionally address and empathize with the human impact of illness.

The process of interacting with patients slowly becomes demystified. Bedside teaching facilitates students to become increasingly confident in speaking to and examining real patients, with the reassurance of a more experienced practitioner at hand. Bedside teaching also opens the mind to the reality of clinical medicine that perhaps cannot be mimicked with an actor. It often takes time to sensitively and accurately get a thorough history from a patient. The balance of being efficient with time, yet establishing a rapport with patients, can be learned. Although some clinical signs and experiences can be simulated, many cannot (e.g. the tactile experience of hepatosplenomegaly). Peters and ten Cate provide some interesting evidence to support this improvement in clinical skills through bedside teaching.

## Finding a role for bedside teaching in the modern era

Students, teachers, and patients value bedside teaching, so how might we achieve more of it? At a university level teaching guidelines should be devised in conjunction with both ward staff, and patient advocacy groups. They could be written to minimize disruption to ward work, and to ensure preservation of patient autonomy. Protected time for bedside teaching should be allocated to teachers. This might be achieved by incorporating bedside teaching into job plans, particularly for junior doctors. Junior doctors are an important and effective pool of teachers [8]. This group have freshly learned clinical examination skills, and are often keen to impart their wisdom.

The development of teaching skills should begin at undergraduate level, with the incorporation of both teaching methodology, and teaching opportunities into the curriculum. Tutorials led by senior medical students have been implemented successfully [9, 10], with similar acceptability to those led by senior staff.

## Progress for change

The ultimate solution is incorporating more bedside teaching into student timetables, and giving doctors more protected teaching time. However, change at a hospital and university level by its nature is slow. The reality for current students is that many will graduate before their universities can provide them with a desirable amount of bedside teaching.

So what interim solution might there be? One effective approach was demonstrated in South East Scotland. Junior doctors and medical students set up a complimentary bedside teaching programme to the main curriculum [11, 12]. It was approved by the medical school, and individual hospital wards. Nothing had to be ‘sacrificed’ from the main curriculum since the programme was complimentary. A training day was set up for the ‘tutors’ to reinforce key teaching principles. Students and junior doctors communicated directly with each other to organize teaching by using web-based forums. Rapid exchange of information in this arena catered for the often unpredictable clinical timetables for both medical students and doctors. This approach was also applied to prescribing and simulation scenario teaching. In all three programmes, the methodology was very effective in increasing learning opportunities. In 2010–2011, 108 junior doctors gave 324 tutorials using this method, with 1,923 cumulative attendances from Edinburgh University medical students. No students missed lectures or tutorials organized by the medical school; these were all additional opportunities.

## Conclusions

The benefits of bedside teaching are clear, and the criticisms have been addressed well by Peters and ten Cate. Students want more, doctors want to deliver more. Change needs to happen at university and hospital level. However, in the meantime, collective action from the students who want to learn, and the doctors who want to teach has the potential to increase bedside teaching.

And more bedside teaching can only be a good thing.
